# SARS-CoV-2/COVID-19: Viral Genomics, Epidemiology, Vaccines, and Therapeutic Interventions

**DOI:** 10.3390/v12050526

**Published:** 2020-05-10

**Authors:** Mohammed Uddin, Farah Mustafa, Tahir A. Rizvi, Tom Loney, Hanan Al Suwaidi, Ahmed H. Hassan Al-Marzouqi, Afaf Kamal Eldin, Nabeel Alsabeeha, Thomas E. Adrian, Cesare Stefanini, Norbert Nowotny, Alawi Alsheikh-Ali, Abiola C. Senok

**Affiliations:** 1 College of Medicine, Mohammed Bin Rashid University of Medicine and Health Sciences, Dubai, UAE; Mohammed.Uddin@mbru.ac.ae (M.U.); tom.loney@mbru.ac.ae (T.L.); Hanan.Alsuwaidi@mbru.ac.ae (H.A.S.); Thomas.Adrian@mbru.ac.ae (T.E.A.); Norbert.Nowotny@mbru.ac.ae (N.N.); 2 The Centre for Applied Genomics, The Hospital for Sick Children, Toronto, ON M5G 0A4, Canada; 3 Department of Biochemistry, College of Medicine and Health Sciences, United Arab Emirates University, Al Ain, UAE; fmustafa@uaeu.ac.ae (F.M.); ahmedh@uaeu.ac.ae (A.H.H.A.-M.); 4 Department of Microbiology & Immunology, College of Medicine and Health Sciences, United Arab Emirates University, Al Ain, UAE; tarizvi@uaeu.ac.ae; 5 Department of Food, Nutrition and Health, United Arab Emirates University, Al Ain, UAE; afaf.kamal@uaeu.ac.ae; 6 Ministry of Health and Prevention, Dubai, UAE; dr.nabeel@yahoo.com; 7 Department of Biomedical Engineering, Healthcare Engineering Innovation Center (HEIC), Khalifa University, Abu Dhabi, UAE; cesare.stefanini@ku.ac.ae; 8 Viral Zoonoses, Emerging and Vector-Borne Infections Group, Institute of Virology, University of Veterinary Medicine Vienna, 1210 Vienna, Austria

**Keywords:** SARS-CoV-2, COVID-19, coronavirus, pandemic, viral genomics

## Abstract

The COVID-19 pandemic is due to infection caused by the novel SARS-CoV-2 virus that impacts the lower respiratory tract. The spectrum of symptoms ranges from asymptomatic infections to mild respiratory symptoms to the lethal form of COVID-19 which is associated with severe pneumonia, acute respiratory distress, and fatality. To address this global crisis, up-to-date information on viral genomics and transcriptomics is crucial for understanding the origins and global dispersion of the virus, providing insights into viral pathogenicity, transmission, and epidemiology, and enabling strategies for therapeutic interventions, drug discovery, and vaccine development. Therefore, this review provides a comprehensive overview of COVID-19 epidemiology, genomic etiology, findings from recent transcriptomic map analysis, viral-human protein interactions, molecular diagnostics, and the current status of vaccine and novel therapeutic intervention development. Moreover, we provide an extensive list of resources that will help the scientific community access numerous types of databases related to SARS-CoV-2 OMICs and approaches to therapeutics related to COVID-19 treatment.

## 1. Introduction

In December 2019, several cases of a new respiratory illness were described in Wuhan, Hubei Province, China. By January 2020, it was confirmed that these infections were caused by a novel coronavirus which was subsequently named SARS-CoV-2, while the disease it caused COVID-19 [1,2]. This novel coronavirus is closely related to the previously described SARS-CoV identified in the 2002–2003 outbreak [3]. The World Health Organization (WHO) recently declared the ongoing SARS-CoV-2 outbreak as a pandemic [1]. To contain the spread of the virus, we are witnessing the implementation of strict measures unprecedented in modern times. Major cities and entire nations have been placed under lockdown with restrictions on travel and gatherings as well as closure of schools and businesses. These measures, along with the closure of international borders and restrictions on international travel have had significant economic impact, resulting in a sharp decline in major financial indices and prompting fears of a global recession.

As the number of confirmed infections and fatalities continue to increase daily, it is crucial to further our understanding of the virus transmission patterns and epidemiology. Despite only a few months into the outbreak, there is a wealth of information emerging on the virus genomic makeup and evolution, and its transcriptomic mapping, including virus–human protein interactions. Such information is urgently needed for the identification of therapeutic targets for intervention and vaccine development, in addition to informing preventive policies and patient care decisions. The primary purpose of this review is to provide an update on the epidemiology, modes of transmission, a summary of the genomics, and transcriptomics of SARS-CoV-2, as well as therapeutic interventions in the absence of a vaccine. Furthermore, we examine how the viral genomics and molecular epidemiology informs therapeutic and vaccine development as well as public health strategies. We have also compiled a resource table outlining the numerous databases related to SARS-CoV-2 whole genome sequencing, transcriptomic map, strain tracing, SARS-CoV-2-human protein–protein interactions, and clinical trials for repurposed drugs and vaccines (Table 1).

## 2. Epidemiology and Transmission of SARS-CoV-2

To date (10 May 2020), over 4 million laboratory-confirmed cases of COVID-19 have been reported worldwide with more than ~279,000 deaths in 187 countries [4]. In most countries, increases in the number of confirmed cases are following an exponential growth trajectory during the early and peak stages of the outbreak. At present, the global case fatality rate of COVID-19 laboratory confirmed cases is ~6.9% ranging from ~0.1% in Singapore to ~16.3% in Belgium [4]. Whilst it is difficult to compare case fatality rates between countries when they are at different stages of the outbreak, variations are most likely due to the scope of population testing, the age structure, and health status of the population, and the health systems within each country. Clinical characteristics of SARS-CoV-2 patients from China [5,6], South Korea [7], and the United States [8] have recently been reported with fever, dry cough, and shortness of breath being the most common clinical presentations. Although the outbreak is evolving, the global data suggest that the number of cases doubled every four days, with ~20% of confirmed COVID-19 patients requiring hospitalization (median hospital stay of 12 days), and 25% of hospitalized patients (~5% of all cases) needing intensive critical care [5,7,8]. The severity and outcome of the disease seem to be highly correlated with the age of onset where more severe forms of COVID-19 were observed for adults ≥ 55 years [5,7,8]. Additionally, an age-dependent fatality rate has been demonstrated with the lowest risk observed among those under the age of 19 (0–0.1%) and 20–54 years (0.1–0.8%); however, the risk of mortality increases incrementally, affecting 1.4–4.9% in the 55–74-year age group, 4.3–10.5% among those aged 75–84 years, with the highest fatality rate of 10.4–27.3% in those aged ≥85 years [5,7,8,9]. Individuals with underlying health issues such as cardiovascular disorders, diabetes, liver, and kidney disease, malignant tumors, or a suppressed immune system, seem to have the severe form of the disease and increased fatality rate [5,7,8,9,10].

Current evidence suggests that SARS-CoV-2 is likely to have a natural origin [11] and is primarily transmitted via inhalation of droplets expelled when an infected patient coughs. Fomite-mediated transmission is another important source of transmission when hands which have touched surfaces contaminated by droplets are used to touch the face, eyes, or nose. Modeling of SARS-CoV-2 spread estimation from multiple studies suggests that the basic reproduction number (R_0_) ranges from 2.2 to 5.7 depending upon the population [12,13] (see Box 1). This reported R_0_ is higher than seasonal influenza, indicating the potential for sustained human-to-human transmission within a population unless strict containment and public health measures are implemented and sustained. As a new coronavirus, there is currently insufficient data to reach a consensus on the potential for seasonality of SARS-CoV-2 transmission since the human population is completely naïve to this virus. Keeping this factor aside, two major factors that may have an influence on seasonality are changes in environmental parameters and human behavior [14]. Specifically, outdoor (e.g., temperature, humidity, sunlight/vitamin D status) and indoor environmental factors (e.g., temperature, humidity, air change rate, etc.) influence both virus transmission parameters (e.g., virus viability, airborne aerosolization, droplet spray, and direct contact) and host defenses (e.g., airway antiviral immune defense and efficiency of nasal and bronchial mucociliary clearance). Although the seasonality has not been confirmed for SARS-CoV-2, there is now accumulating evidence that climate variables might play a role in transmission [15,16].

The stability of SARS-CoV-2 in aerosols as well as on surfaces has been evaluated [17]. Findings from a series of well-controlled experiments revealed that the virus remained infectious in aerosols throughout the duration of the experiment (3 h; median half-time of 1.1–1.2 h) [17]. Additionally, in relation to surfaces, SARS-CoV-2 was found to be most stable on plastic and stainless steel with infectious virus detected up to 72 h post-application and no infectious virus was found on copper or cardboard after 4 and 24 h, respectively [17]. In this experimental model, SARS-CoV-2 exhibited similar stability to SARS-CoV. Therefore, the differences in the epidemiological trends of the 2002–2003 SARS-CoV outbreak and the ongoing SARS-CoV-2 pandemic are more likely due to other factors such as high viral loads in the upper respiratory tract and the potential for individuals infected with SARS-CoV-2 to shed and transmit the virus whilst asymptomatic [17,18,19]. Overall, the findings indicate that continued aerosol and fomite transmission (see Box 1) of SARS-CoV-2 is highly plausible as the virus remains infectious in droplets for numerous hours and on surfaces for up to three days [17,20]. This has now raised the concern that airborne transmission might be occurring [17,20,21,22], though epidemiological evidence minimizes the relevance of such transmission to disease spread [23,24].

Box 1 **SARS CoV-2-related Definitions.**

- SARS-CoV-2: Severe acute respiratory syndrome coronavirus 2
- COVID-19 or Covid-19: Corona virus disease, 2019. COVID-19 is the official name of the disease manifested by SARS-CoV-2.
- R_0_: Reproduction number that defines the number of secondary cases that will be produced by a single infectious index case in a population that is fully susceptible to the disease. For example, a R_0_ of 2 means that, on average, one primary index case would infect two other people, generating two secondary cases. Continuous horizontal (human-to-human) transmission will occur if R_0_ is above the critical threshold of one.
- Fomite Transmission: A fomite is any inanimate object (i.e., surface) when contaminated with or exposed to infectious agent, can serve as a source to transmit the agent into a new host.
- Non-Pharmacological Interventions (NPIs): NPIs are evidence based, non-invasive, mostly policy/regulation driven interventions on human health. NPIs (i.e., physical [“social”] distancing) can be very effective to contain viral shedding.

## 3. Genomics of SARS-CoV-2

**Figure 1:**
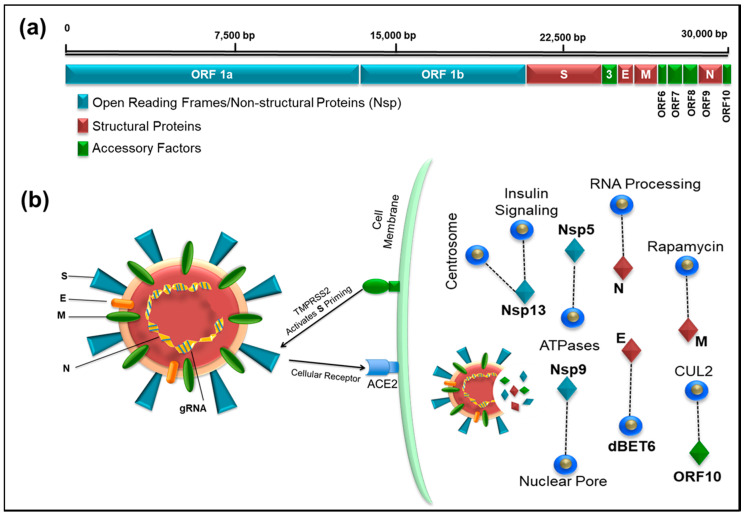
(**a**) Illustration of the full-length genome of SARS-CoV-2 showing the location of open reading frames 1a and 1b encoding the Non-structural proteins, Nsp (blue), structural proteins (brown), and accessory factors (green). The numbers on top refer to the genomic RNA; (**b**) schematic representation of the SARS-CoV-2 virus particle and its interaction with its host cellular receptor, ACE2. The infection pathway is shown where after docking of the virus particle on cell surface, the TMPRRSS2 cellular protease activates the viral protein S allowing entry of SARS-CoV-2 into human cells. The protein coded by the viral genes and some of the notable interactions (dashed line) with other host proteins are shown that can potentially be targeted by drugs (blue circles).

SARS-CoV-2 is a β-coronavirus similar to the viruses that cause SARS (severe acute respiratory syndrome) and MERS (Middle East respiratory syndrome). Human coronaviruses are not new and have been identified in the population since the late 1960s, causing mild symptoms similar to common colds [25]. Of the seven virus species known, four infect the upper respiratory tract and cause mild symptoms, while three are associated with the lower respiratory tract, causing severe disease, including SARS-CoV, MERS-CoV, and now SARS-CoV-2 (reviewed in Lu et al.) [26]. Like other coronaviruses, SARS-CoV-2 is an enveloped, single-stranded, positive-sense RNA virus with a non-segmented genome ~30 kb in size [11,27] (Figure 1). The viral genome codes for 16 non-structural proteins (Nsps) required for virus replication and pathogenesis, four structural proteins, including envelope (E), membrane (M), nucleocapsid (N), and spike (S) glycoprotein important for virus subtyping and response to vaccines, and nine other accessory factors [27,28] (Figure 1). The first SARS-CoV-2 genome was published on 24 January 2020, only a few weeks into the outbreak [29], and exhibited genomic and phylogenetic similarity to SARS-CoV, particularly in the S gene and receptor-binding domain (RBD), indicating the capability of direct human-to-human transmission. The genomic sequence of SARS-CoV-2 shows that, although it is 75–80% identical to SARS-CoV [3,11], it is even more closely related to several bat coronaviruses, in particular the Bat SARS-related coronavirus SARSr-CoV RaTG13 [29]. Phylogenetic analyses of SARS-CoV-2 genomes have identified bats as the primary reservoir of SARS-like coronaviruses [30] displaying high sequence similarity (96.2%) between BatCoV and SARS-CoV-2 genomes [31]. Sequence analysis of the viral spike protein further suggests new mutations in its RBD determine not only the host range but also the cellular tropism of the virus [2,32,33,34]. Interestingly, a similar observation was made in viruses from pangolin SARSr-CoVs, one of the putative intermediate host species that may have been used by SARS-CoV-2 for its species jump into humans [35]. A few months prior to the emergence of SARS-CoV-2, the Pangolin-CoV whole genome was sequenced from a dead Malayan Pangolin (*Manis javanica*) that showed 91.02% and 90.55% identical genome sequences to SARS-CoV-2 and BatCoV RaTG13, respectively [36]. The sequence analysis also revealed that the S1 protein of Pangolin-CoV was much more closely related to SARS-CoV-2 than to RaTG13. Whilst these findings suggest Pangolin species as a reservoir of coronaviruses, the analysis does not prove the potential of Pangolin as the intermediate host of SARS-CoV-2.

Sequences of SARS-CoV-2 have now been reported from many parts of the world, and these data have proved useful in tracking the global spread of the virus [37] (see Table 1 for resources related to SARS-CoV-2 genomics). For example, an initial analysis of 103 SARS-CoV-2 genomes identified two major subtypes (which were designated L and S) that are well-defined by two different single nucleotide polymorphisms (SNPs) [35]. RNA viruses tend to harbour error-prone RNA-dependent RNA polymerases which make occurrence of mutations and recombination events rather frequent [38,39,40,41]. This might play a role in the evolution of SARS-CoV-2. Within Wuhan, China, the L type was found in ~70% of cases and was observed to be the more aggressive and contagious form compared to the original S type [35]. The virus has further mutated and expanded into numerous strains and clusters (Table 1) [42,43,44]. The geographical diversity of different strains may help correlate COVID-19-related severity, mortality rate, and treatment options. For example, using a phylogenetic network analysis approach on 160 full-length genomes, a recent study has shown that the virus seems to be evolving into three distinct clusters, with A being the ancestral type closest to the bat genome and found mostly in Americas and Europe along with the C type, while B being the most common type in East Asia [45]. Genomic epidemiology of SARS-CoV-2 should also shed light on the origins of regional outbreaks, global dispersion, and epidemiological history of the virus (Table 1) [11,35]. More importantly, in case of an inability to diagnose infections empirically due to the speed of epidemics or lack of test kits, such as the case with COVID-19, genomic epidemiology could be used to estimate virus rate of replication in the population as well as burden of infection, allowing healthcare professionals to make urgent policy decisions appropriately. There is ongoing work geared towards mapping the spread of different SARS-CoV-2 strains across the world.

## 4. Transcriptomic Map and SARS CoV-2-Human Protein–Protein Interactions to Identify Drug Targets

The transcriptome profile of SARS-CoV-2 isolated from COVID-19 patients has recently been constructed using both ”long read DNA/RNA (Nanopore) sequencing” and “sequencing by synthesis” techniques [27] (see Table 1 for SARS-CoV-2 sequencing and OMICs related resources). Direct RNA sequencing (without requiring reverse transcription) has further allowed detection of RNA modifications on the genomic RNA (Table 1). By combining both sequencing and RNA modification data, scientists in South Korea have identified 41 potential RNA modification sites that could be important for virus replication and its associated pathogenesis [27]. The transcriptomic insights should further provide a better understanding of the viral life cycle and its virulence.

After cell entry, the virus RNA transcript produces nonstructural proteins (Nsp1 through Nsp16) using two open reading frames (ORF1a and 1b, Figure 1a). A recent study on protein–protein interaction mapping using mass spectrometry identified 332 SARS-CoV-2-human protein interactions, including 69 interactions that can be targeted by existing FDA-approved drugs [28] (Table 1). We observed one interesting similarity in both the transcriptomic and proteomic studies, where the last reading frame (ORF10) expression was extremely low. Although the transcriptomic study questioned the annotation of ORF10 due to extremely low RNA expression, the proteomic analysis identified strong interaction of ORF10 with CUL2 complex, an E3 ubiquitin-protein ligase complex that mediates ubiquitination of target proteins [27,28]. This suggests that the virus may be able to subvert this complex and use it for degradation of host restriction factors that limit virus replication, making it a good target for drug development against the virus.

In humans, the *ACE2* gene encodes the angiotensin-converting enzyme-2. Evidence from recent studies suggests that ACE2 is the host receptor for the novel SARS-CoV-2 similar to SARS-CoV [46,47]. The binding of SARS-CoV-2 to the ACE2 receptor (via the S protein) [47] is 10–20-fold higher compared to SARS-CoV, which may be one of the reasons for the higher human-to-human transmission of SARS-CoV-2. The binding between SARS-CoV-2 and ACE2 has been confirmed by multiple recent independent studies [28,46]. ACE2 is primarily found in the lower respiratory tract of humans on epithelial cells lining the lung alveoli and bronchioles as well as the endothelial cells and myocytes of pulmonary blood vessels, partly explaining the severe respiratory syndrome associated with these viruses [48]. Its expression in the nasal epithelial cells of the upper respiratory tract has recently been confirmed using single cell RNAseq data, suggesting another reason for the high transmission rates of the virus [49]. ACE2 is also found on the enterocytes in the small intestines, which may further explain the gastrointestinal symptoms associated with the viral infection as well as its detection in faeces [50]. In a recent study, it has been shown that the *ACE2* gene displays single nucleotide polymorphims with differential allele frequency accross the globe [51]. The allele frequency for the host gene was also shown to be different between males and females.

The viral spike (S) protein is responsible for viral entry into susceptible cells by interacting with the ACE2 receptor [46]. This process requires “priming” of the S protein by the host transmembrane serine protease 2 (*TMPRSS2*) which cleaves the S protein into two functional subunits: S1 and S2. The S1 subunit then is able to interact with the ACE2 receptor, while the S2 subunit facilitates viral fusion with the host cell membrane, allowing virus entry into the target cell [46] (Figure 1a). The current knowledge of the cellular infection pathway involving ACE2 and TMPRSS2 thus provide good candidates for therapeutics, such as antibodies that can interfere with virus attachment and fusion with target cells (such as protease inhibitors).

## 5. Diagnosis of COVID-19

As the COVID-19 pandemic continues to spread rapidly, there is a growing demand for rapid point-of-care testing of the virus. The current gold standard for diagnosing COVID-19 depends upon detection of the viral genetic material (RNA) in a nasopharyngeal swab or sputum sample. While this technique is sensitive and can detect the virus earlier in the infection, it requires polymerase chain reaction (PCR), a technology that amplifies the amount of genetic material to detectable levels and takes several hours to perform [52]. In recent weeks, rapid molecular tests using automated platforms have received fast-track approvals from regulatory authorities. These are high throughput automated tests with a turnaround time of 45–60 min.

To detect newer mutated viruses, it is essential to apply next generation sequencing to identify the viral genome with specific mutations. Currently, “sequencing by synthesis” technique (by Illumina Inc. San Diego, CA, USA) and “long read sequencing” (by Oxford Nanopore Technology, Oxford, UK) are being used to identify viral genomes at single-base resolution levels [11,27,35]. Although nanopore sequencing technology has a higher error rate, this flaw can be complemented with the use of other sequencing techniques, such as sequencing by synthesis. However, nanopore sequencing technology might have an advantage over other sequencing platforms due to its small and compact size, allowing flexibility of conducting RNA sequencing in the field in remote locations that lack full-fledged accredited reference laboratories.

Besides targeting the genome, another diagnostic approach for SARS-CoV-2 aims at detecting antibodies produced by the patient’s immune system against the virus. Scores of such “antibody” tests have been reported over the past few months for SARS-CoV-2 [53]; however, confirmation of validity of several of these assays remains underway. Although the antibody associated tests are faster, their use for diagnosis is limited by the fact that it usually takes several days and up to two weeks after an infection takes place for antibodies to be detectable. Therefore, antibody-based testing is not a reliable method to diagnose COVID-19; however, they may be useful for population-based testing to estimate the proportion of the population with immunity (if antibodies are a marker of immunity) and identifying susceptible individuals. Such information may also be useful for public health measures, including return-to-work protocols and social segregation of susceptible individuals. A third type of testing relies on detecting viral proteins (antigens) likely to be useful since they do not depend on a detectable rise in patient-produced antibodies [54]. Globally, several companies are working on developing such rapid antigen-antibody-based and CRISPR-Cas12 based assays which have received rapid emergency use authorization by respective regulatory agencies [55]. Unfortunately, up to now, the reliability of point-of-care antigen and antibody tests is limited, mainly due to cross-reactions with other coronaviruses. The diagnostic gold standards are still various RT-qPCR assays.

A comprehensive list of SARS-CoV-2 diagnostic assays (both molecular and immunological) that have been commercialized and those under developments globally can be found at: https://www.finddx.org/covid-19/pipeline/.

## 6. Development of Vaccines and Experimental Therapeutic Interventions for SARS-CoV-2

### 6.1. Vaccine Development 

Many efforts are in progress to produce a vaccine for SARS-CoV-2. The approaches include the classical inactivated and attenuated vaccines (7 teams are working on this with two inactivated vaccines in clinical trials), the protein subunit and virus like particle vaccines (VLP) (28 teams on the subunit vaccines, mostly on the spike protein and 5 on VLPs), viral vector-based vaccines (~25 teams with one in clinical trial), as well as the newer DNA- and RNA-based vaccines (20 teams with one of each type in clinical trials) [56]. Each approach has its own advantages and disadvantages and all approaches are being developed simultaneously to come up with an effective vaccine (reviewed in Amanat and Krammer, 2020) [57].

Among the four structured proteins of the virus, the spike protein is considered the most promising for vaccine development since: (i) it is common to different coronaviruses encountered, and (ii) it is exposed to an individual’s immune system, allowing the body to make an immune response against it and remember it for future protection. Furthermore, such a vaccine can prevent infection since it would inhibit virus entry into susceptible cells. Due to previous experience with vaccine development for SARS in 2003 (against SARS-CoV), scientists have had a head start in using the S protein for vaccine development and some vaccines have entered human clinical trials, while others are on their way [57,58].

So far, the first vaccine to enter into clinical trials is the mRNA-1273 vaccine (ClinicalTrials.gov: NCT04283461). It is a novel RNA-based vaccine which uses part of the spike protein genetic code embedded in special lipid-based nanoparticles for injection into the body [59]. It has been developed at lightning speed (within 45 days of publication of the first viral genome) by Moderna Therapeutics (Cambridge, MA, USA) who was already working on SARS-CoV and MERS-CoV vaccines which were adapted to SARS-CoV-2. After having shown potential in animal testing, the first phase I clinical trial of this vaccine started on 16 March 2020 in collaboration with the NIH on 45 healthy individuals between the ages of 18–55 years [57]. However, in addition to the novelty of this vaccine, even if the clinical trials are successful, it will be quite some time before it can be available to the population due to pipeline, capacity building, and regulatory issues. Several other mRNA-based vaccines (e.g., by CureVac (Tübingen, Germany), BNT162 by BioNTech (Mainz, Germany) and Pfizer (New York, NY, USA)) are in different stages of development. For instance, the BioNTech mRNA vaccine (Mainz, Germany) encapsulates the nucleic acid in special 80 nm ionizable, glycol-lipid nanoparticles and clinical testing is expected commence shortly [59].

Another vaccine that has entered clinical trials in China has been developed by CanSino Biologics (Tianjin, China), the company that also has developed a vaccine for Ebola. Also based on the S protein, the vaccine (Ad5-nCoV) is based on their adenovirus vaccine platform, and is undergoing phase I clinical trials in healthy individuals between 18–60 years of age in Wuhan, China- (ClinicalTrials.gov: NCT04313127) [60].

Other than these, there has been an acceleration in developing other novel vaccine approaches and therapeutic interventions to combat viral infection [57,59,60]. For example, Inovio Pharmaceuticals’ INO-4800 (Plymouth Meeting, PA, USA) is a DNA-based vaccine using the *spike* gene. Funded by the Bill and Melinda Gates Foundation, the vaccine has already entered phase I clinical trials for intradermal delivery using electroporation. Codagenix, in collaboration with Serum Institute of India, has used a reverse strategy to create a live-attenuated vaccine in which viral sequences have been changed by swapping its optimized codons with non-optimized ones to weaken the virus. Since live-attenuated vaccines have a higher chance of success, in anticipation, large scale manufacture of this vaccine has already started in India. Shenzhen Geno-Immune Medical Institute, on the other hand, has two vaccines in clinical trial based on dendritic cells and antigen presenting cells modified by lentiviral vectors expressing portions of the SARS-CoV-2 genome as “minigenes”. Johnson and Johnson (New Brunswick, NJ, USA) and Altimmune Inc. (Gaithersburg, MD, USA) are developing intranasal, recombinant adenovirus-based vaccines to stimulate the immune system. Which one of these strategies will be most efficacious is hard to predict and hopefully some of them will be successful; thus, major international vaccine funding agencies are supporting a multitude of innovative efforts to find the best ones for eventual large-scale production. An extensive list of vaccines is under development including those highlighted above, their current status can be found at the Milken Institute COVID-19 Treatment and Vaccine Tracker available at: https://milkeninstitute.org/sites/default/files/2020-03/Covid19%20Tracker%20032020v3-posting.pdf.

### 6.2. Experimental Therapeutic Interventions

#### 6.2.1. Convalescent Plasma (CP) Therapy

This is a classic adaptive immunotherapy that has been applied to many infectious diseases for more than a century for prevention and treatment. CP has been shown to be successful over the last two decades against SARS, MERS, and H1N1 infection [61,62,63]. In this therapy, plasma (with neutralizing antibodies) is extracted from a donor who has recovered from the infection, followed by its administration to infected patients. Preliminary work describing administration of CP to severe COVID-19 patients have reported significant improvement and large scale clinical trials are ongoing [64,65]. In addition to this classical approach, others are trying to identify and characterize specific antibodies generated by recovering patients to determine if these can be used to develop functional antibodies as a treatment for COVID-19 [59,66]. For example, AbCellera, a Canadian biotech (Vancouver, BC, Canada), has discovered >500 unique antibodies from sera of a convalescent COVID-19 patient, and in partnership with Eli Lilly, is developing purely human IgG1 mAbs-based treatments for coronavirus infection. Similarly, InflaRx (Jena, Germany) and Beijing Defengrei Biotechnology (Beijing, China) are using human IgG1 mAbs against complement factor 5a as therapy since C5 has been observed to be the major cause of tissue injury in patients. Such antibodies have already been approved for clinical trials in China. Other novel therapies for COVID-19 include an effort by Alnylam Pharmaceuticals (Cambridge, MA, USA) that has developed a technology for delivering aerosolized siRNAs against SARS-CoV-2 directly to lungs which is being tested both in vitro and in vivo. Similarly, nanoviricides are being created in another approach in which the S protein is chemically attached to “virucidal nanomicelles”.

#### 6.2.2. Soluble Human Angiotensin-Converting Enzyme 2 (ACE2)

ACE2 is the host receptor for SARS-CoV-2 infection that interacts with the viral spike protein to gain entry into human cells; therefore, it has been suggested that hindering this interaction could potentially be used as an effective treatment in COVID-19 patients [67]. Consistent with this hypothesis, a recent in vitro study has shown that clinical-grade human recombinant soluble ACE2 (hrsACE2), but not mouse soluble ACE2, could curtail replication of SARS-CoV-2, resulting in reduced viral loads drastically in Vero cells in a dose dependent manner [68]. Furthermore, they go on to show that hrsACE2 could inhibit virus infection of human engineered blood vessel and kidney organoids. These are promising observations and open a new window of opportunity to use hrsACE2 to prevent SARS-CoV-2 infection at a very early stage by blocking its entry into the target cells, thus potentially protecting patients from lung injury.

Novel therapeutic interventions under development, including those highlighted above, as well as their development status can be followed at the Milken Institute COVID-19 Treatment and Vaccine Tracker: (https://milkeninstitue.org/sites/default/files/2020-03/Covid19%20Tracker%20032020v3-posting.pdf).

## 7. Drug Repurposing for COVID-19

Given the need to find effective treatment for symptomatic patients, the approach of repurposing old drugs with antiviral properties and agents approved or under investigation for other viral infections has been adopted. In the abscence of a vaccine, WHO recently launched the SOLIDARITY trial which is an international clinical trial to address this challenge. The drugs included in this trial are lopinavir and ritonavir, lopinavir and ritonavir plus interferon beta as well as chloroquine, and remdesivir. The roles of existing antiretroviral drugs and pathways in COVID-19 treatment are as follows:

- **Lopinavir (LPV)-Ritonavir (RTV) combination (Kaletra):** This is an FDA-approved drug for HIV-1 treatment. Lopinavir is a protease inhibitor that inhibits virus particle maturation, a late step in HIV-1 replication, while ritonavir helps boost the activity of lopinavir by inhibiting CYP3A enzymes that slows down the rate at which lopinavir is broken down in the liver [69]. Findings from in vitro and animal studies against both SARS and MERS indicate its potential for COVID-19 treatment [69,70,71,72]. Lopinavir-Ritonavir has been used either on its own or in combination with either alpha interferon (China) or chloroquine/hydroxychloroquine (South Korea) for COVID-19 treatment with some success [73,74]. However, new data from China cast doubt on the beneficial effect in seriously ill COVID-19 patients [75]. Thus, results from additional clinical trials are needed to establish the efficacy of this treatment for COVID-19 which are currently underway.
- **Favipiravir (Favilavir or Avigan):** Favipiravir (FPV) is an RNA-dependent RNA polymerase inhibitor developed by Fujifilm Toyama Chemical in Japan that is safe and has been effective in other viral infections, including influenza [76,77]. It has now been shown to be useful against SARS-CoV-2 in initial clinical trials conducted in Wuhan and Shenzhen [78]. In this study, the effects of FPV versus LPV/RTV were compared during the treatment of COVID-19 patients. The FPV-treated patients demonstrated much better therapeutic response especially with regard to faster viral clearance and improvement rate in chest imaging. Based on these encouraging results, favipiravir has been approved by the National Medical Products Administration of China as the first anti-COVID-19 drug in the country [66].
- **Chloroquine/Hydroxychloroquine:** Chloroquine is an inexpensive drug for the treatment of malaria and features on the WHO list of essential medicines. It is also used as an anti-inflammatory agent for the treatment of autoimmune diseases. Chloroquine is thought to inhibit virus replication by increasing endosomal pH as many viruses such as Ebola and Marburg that require the acidic environment of the endosome for successful replication [79,80,81]. However, a recent study showed that the anti-inflammatory effects of chloroquine are mediated by upregulation of the cyclin-dependent kinase inhibitor, p21 [82]. In vitro studies have shown its potent antiviral effect against the SARS-CoV-2 [83]. A multicenter clinical trial in China has reported efficacy with amelioration of exacerbation of pneumonia and acceptable safety margin with use of chloroquine for treatment of COVID-19 [10]. Hydroxychloroquine is an analogue of chloroquine which is more stable with better clinical safety profile and has anti-SARS-CoV-2 activity. It has been shown to quicken recovery and clearance of the virus in COVID-19 patients and used successfully in combination with the macrolide antibiotic azithromycin [84]. A recent clinical trial, however, has shown disappointing results with the combination of azithromycin with hydroxychloroquine in critically-ill COVID-19 patients [85], suggesting that larger studies with controlled design are needed before conclusive recommendations can be made for chloroquine/hydroxychloroquine in the treatment of COVID-19. Interestingly, chloroquine and hydroxychloroquine are zinc ionophores and zinc has been shown to inhibit RNA-dependent RNA polymerase enzyme of coronaviruses [86,87]. Thus, one reason for the limited success of some of these clinical trials could be due to absence of zinc supplementation which may be necessary to observe the therapeutic effects of these drugs on SARS-CoV-2 and other RNA virus infections [88].
- **Remdesivir (GS-5734):** Remdesivir is a nucleotide analogue prodrug with broad spectrum antiviral activity against many RNA viruses [89]. Like Favipiravir, it blocks RNA-dependent RNA polymerase, an enzyme that replicates the viral genome, inhibiting an early step in virus replication, compared to protease inhibitors that target the late steps of virus replication [90,91]. It has also shown to inhibit replication of MERSCoV, SARS-CoV, and SARS-CoV-2 in animal models [83,89,92,93]. So far, it has been used as an investigational drug for the treatment of Ebola, MERS-CoV, and SARS-CoV2, and other RNA viruses, but has not been approved for any disease [83,89,92,93]. In a compassionate use of remdesivir in a cohort of patients hospitalized for severe COVID-19, the developers of the drug (Gilead Sciences, City, US State abbrev., USA) reported clinical improvement in 68% (36 of 53) of patients [94]. The first randomized, double-blind, placebo-controlled, multicenter clinical trial of remdesivir in 237 patients from Hubei, China, has just been published [95]. Unfortunately, it did not show statistically meaningful clinical benefits except for numerical reduction in time to clinical improvement [95]. Furthermore, treatment with remdesivir had to be stopped early in some patients because of undesirable effects in 12% patients versus 5% patients on placebo. Similar results have been announced from the first US clinical trial of the drug at the time of this writing, which are still unpublished. Further results are awaited on multiple clinical trials of remdesivir in several countries for more conclusive guidelines on its use in COVID-19 patients.
- **SNG001:** SNG001 is an inhaled experimental drug (interferon beta) developed by the UK biotech firm Synairgen. The ability to inhale the drug will allow the patients to “self-administer” it by using a small hand-held nebulizer. It was developed for the severe lung disease chronic-obstructive pulmonary disorder (COPD), but, due to the current COVID-19 crisis, it has been fast-tracked for use in a 100-patient phase II clinical trial (EudraCT2020-001023-14) in the UK (https://adisinsight.springer.com/drugs/800024480), the results of which are awaited.
- **Tocilizumab:** Tocilizumab is a humanized monoclonal antibody against the interleukin-6 receptor (IL-6R) that is approved by FDA to treat patients with rheumatoid arthritis, systemic juvenile idiopathic arthritis, and giant cell arteritis [96]. IL-6 has been shown to be a key mediator of cytokine release storm (CRS) observed in critically ill COVID-19 [96]. Therefore, it has been proposed as a potential therapy to treat such patients [97]. Thus, Tocilizumab has recently been used as an immunosuppressive agent during CRS observed in severely ill COVID-19 patients in China and Italy with promising results [98,99]. COVID-19 patients treated with Tocilizumab in China showed marked improvement indicating that Tocilizumab potentially could be very effective in treating patients with severe infection. Consistent with this, administration of tocilizumab in a COVID-19 patient with pneumonia in Italy showed favorable changes of CT findings within 14 days of treatment [100]. It is turning out to be a promising therapy to treat severely ill COVID-19 patients.
- **Kinases:** p21-activated protein kinases (PAKs) are cytosolic serine/threonine protein kinases downstream of small (p21) GTPases, including members of the Cdc42 and Rac families. Multiple studies have shown that the major pathogenic kinase in this group, PAK1, plays an important role in the entry, replication and spread of several important viruses, including influenza and HIV [101,102]. Coronaviruses exploit macropinocytosis to gain entry into cells and this process has been shown to be dependent on PAK1 activity [103,104]. Targeting of PAK1 to prevent micropinocytosis has been implicated for therapeutic intervention [105]. This strongly suggests that PAK1-inhibitors could be valuable for the treatment of COVID-19 infection. PAK-1 inhibitors include caffeic acid and its ester, propolis, ketorolac, and triptolide. Unfortunately, all these have problems with solubility and cell penetration. However, newer PAK-1 inhibitors, such as 15K (the 1,2,3-triazolyl ester of ketorolac, that is 500 times more potent at inhibiting PAK1 than the parent compound [106], minnelide (in which a hydroxyl group of triptolide is phosphorylated, boosting its water-solubility over 3000 times [107], and frondoside A [108] are much more potent and may be of value in suppressing the effects of this virus.

## 8. Non-Pharmacological Interventions

At present, there are no vaccines or specific pharmacological interventions available to contain the horizontal transmission of SARS-CoV-2. Moreover, effective COVID-19-specific pharmaceutical interventions and vaccines are not expected to be available for 3–12 months. Therefore, the most effective public health response to the ongoing outbreak is to implement non-pharmacological interventions (NPI) such as early case identification and isolation, vigilant contact tracing of potential secondary cases, travel restrictions and bans, stringent contact reductions, physical (“social”) distancing, improved hygiene, and regular hand washing. Such an approach requires closure of non-essential public spaces, services and facilities, a transition to digital learning modalities for educational institutions, and self-isolation/work from home initiatives for businesses. Modelling estimates indicate that integrated NPIs are likely to achieve the strongest and most rapid effect on lowering the reproductive number and slowing the rate of viral transmission, if implemented early in the outbreak [109]. These NPIs are interim measures as the quest for better understanding of the viral genomics continues and the information garnered unlocks the doors for development of effective therapeutic interventions and vaccines.

## 9. Future Directions for COVID-19 Research

The global efforts to contain the COVID-19 pandemic are primarily aimed to reduce the number and rate of infections, minimize the excessive burden on healthcare systems, and reduce the social and economic impact of the pandemic. These efforts will provide the much-needed respite during the period required for the development, testing, and approval of an effective vaccine. Until vaccines are available, it is likely that non-pharmacological interventions will remain the primary line of defense to contain this pandemic. Therefore, accurate and up-to-date data on the daily number of new cases and the case characteristics can inform modeling of future projections of new cases and planning for anticipated healthcare capacity. Timely and accurate national data on hospital bed and intensive care capacity along with daily census is essential for such planning. National vaccination policies may also impact the severity of the pandemic as it has been hypothesized that universal Bacillus Calmette-Guérin (BCG) childhood vaccine may influence the transmission patterns of SARS-CoV-2 as well as COVID-19 morbidity and mortality [110,111].

It is certain that COVID-19 will have a significant global impact on the social, cultural, and economic infrastructures that are envisaged to be long lasting and may take many years to recover. Healthcare systems should consider integrating effective regulatory measures to tackle future pandemics. This crucial lesson was learnt by countries which experienced the previous SARS-CoV outbreak and informed the response to this pandemic in Hong Kong, Singapore, and Taiwan, for example. Genomic characterization will have implications related to pathogenicity, transmissibility, and response to therapy of the viral isolates for local and global populations. Understanding the genetic makeup of the viral strains is also critical for drug discovery and designing of effective vaccines. To better prepare for the next global pandemic, application of artificial intelligence (AI) should be evaluated to predict and track infections before the outbreak happens. Bluedot Inc., a Canadian AI company for infectious diseases, flagged unusual infection related activity in Wuhan, China and reported the spread nine days before WHO officially declared the outbreak [112]. In this era of emerging viral infections, the global community must work together and deploy the very best of its technological resources to address the current pandemic and ensure preparedness for future outbreaks.

## Figures and Tables

**Table 1:** Resources related to genomics, transcriptomics and phenotypes.

Category	Data Type	Database
SARS-CoV-2 Genome Sequencing Data	DNA Sequencing Data	https://www.ncbi.nlm.nih.gov/genbank/sars-cov-2-seqs/
SARS-CoV-2 Transcriptomic Map	RNA Sequencing Data	Open Science Framework: accession number doi:10.17605/OSF.IO/8F6N9
SARS-CoV-2 and Human Protein Interactions	Mass Spectrometry Raw Data	http://proteomecentral.proteomexchange.org/cgi/GetDataset?ID=PXD018117
SARS-CoV-2 Strains	Genomic Epidemiology	https://nextstrain.org/ncov https://www.gisaid.org/
The COVID-19 Host Genetics Initiative	Host Genetics Data (GWAS, WES, WGS)	https://www.covid19hg.org/
COVID-19 Cell Atlas	Single cell transcriptomics data	www.covid19cellatlas.org
List of Clinical Trials	Clinical Trial Related Information	https://clinicaltrials.gov/ct2/home
